# Is Guillain-Barrè syndrome triggered by SARS-CoV-2? Case report and literature review

**DOI:** 10.1007/s10072-020-04553-9

**Published:** 2020-07-09

**Authors:** Edoardo Agosti, Andrea Giorgianni, Francesco D’Amore, Gabriele Vinacci, Sergio Balbi, Davide Locatelli

**Affiliations:** 1 Division of Neurosurgery, Department of Biotechnology and Life Sciences, University of Insubria, Via Guicciardini, 9, 21100 Varese, Italy; 2 Department of Neuroradiology, ASST Sette Laghi, University of Insubria, Varese, Italy; 3 Department of Radiology, University of Insubria, Varese, Italy

**Keywords:** SARS-CoV-2, COVID-19, Guillain-Barrè, AIDP, Neuropathy, Para-infectious

## Abstract

**Background:**

Severe acute respiratory syndrome coronavirus 2 (SARS-CoV-2) is the infectious agent responsible for coronavirus disease 2019 (COVID-19). Respiratory and gastrointestinal manifestations of SARS-CoV-2 are well described, less defined is the clinical neurological spectrum of COVID-19. We reported a case of COVID-19 patient with acute monophasic Guillain-Barré syndrome (GBS), and a literature review on the SARS-CoV-2 and GBS etiological correlation.

**Case Description:**

A 68 years-old man presented to the emergency department with symptoms of acute progressive symmetric ascending flaccid tetraparesis. Oropharyngeal swab for SARS-CoV-2 tested positive. Neurological examination showed bifacial nerve palsy and distal muscular weakness of lower limbs. The cerebrospinal fluid assessment showed an albuminocytologic dissociation. Electrophysiological studies showed delayed distal latencies and absent F waves in early course. A diagnosis of Acute Inflammatory Demyelinating Polyradiculoneuropathy (AIDP) subtype of GBS was then made.

**Conclusions:**

Neurological manifestations of COVID-19 are still under study. The case we described of GBS in COVID-19 patient adds to those already reported in the literature, in support of SARS-CoV-2 triggers GBS. COVID-19 associated neurological clinic should probably be seen not as a corollary of classic respiratory and gastrointestinal symptoms, but as SARS-CoV-2-related standalone clinical entities. To date, it is essential for all Specialists, clinicians and surgeons, to direct attention towards the study of this virus, to better clarify the spectrum of its neurological manifestations.

## Background

Severe acute respiratory syndrome coronavirus 2 (SARS-CoV-2) is the infectious agent of coronavirus disease 2019 (COVID-19). Starting from the first case recorded in Wuhan (China) in December 2019, SARS-CoV-2 quickly spread around the world, leading the World Health Organization to declare pandemic [1]. To date, June 6th, 2020, the confirmed cases in the world are around 6.6 million and more than 390,000 deaths [2].

COVID-19 is a systemic infection that usually presents with fatigue and fever. The most frequent symptoms described are respiratory and gastrointestinal [3]. However, neurological complications have recently been reported, including dizziness, headache, febrile seizures, myalgia, encephalopathy, encephalitis, stroke, and acute peripheral nerve diseases [4, 5].

Some cases of Acute Motor and Sensory Axonal Neuropathy (AMSAN) and acute inflammatory demyelinating polyradiculoneuropathy (AIDP) subtypes of Guillain-Barré syndrome (GBS) have recently been described. GBS is an acute immune-mediated polyradiculoneuropathy often related to previous infectious exposure [6–8]. Clinically, GBS is characterized by limbs or cranial-nerve weakness, loss of deep tendon reflexes, sensory, and dysautonomic symptoms due to peripheral nerves and root demyelination and/or axonal damage [9]. About 60% of all GBS are preceded by respiratory or a gastrointestinal [10].

We reported a case of COVID-19 patient with AIDP subtype of GBS, associating a literature review on the SARS-CoV-2 and GBS etiological correlation.

## Case description

On April 26th, 2020, a 68-year-old man presented to the emergency department with symptoms of acute progressive symmetric ascending flaccid tetraparesis. Patient medical history included dyslipidemia, benign prostatic hypertrophy, hypertension, and abdominal aortic aneurysm in follow-up. Ten days before admission, dry cough associated with fever, dysgeusia, and hyposmia appeared. Neurological manifestations started 5 days later with progressive acute weakness of distal lower extremities.

On admission, oxygen saturation was 96% on room air, with a respiratory rate of 17 breaths/min, and the body temperature was 37.2 °C. Chest computed tomography highlighted a bilateral basilar ground glass opacity, with oropharyngeal swab positive for SARS-CoV-2 on reverse transcriptase-polymerase chain reaction (RT-PCR) assay. No pathological findings were auscultated on pulmonary objective examination.

The patient was then isolated and antiviral drugs have been started. Neurological examination showed bifacial nerve palsy (House-Brackmann grade 3) and muscular weakness, with a Medical Research Council scale of 1/5 in proximal and 2/5 in distal of the lower limbs. The osteo-tendon reflexes were hypoactive with bilateral areflexia to the Achilles tendons. No sensory deficit was recorded. Upper motor neuron disorder or meningeal irritation signs have not been found.

Baseline laboratoristic analysis showed thrombocytopenia (101 × 10^9^/L, reference value: 125–300 × 10^9^/L) and lymphocytopenia (0.48 × 10^9^/L, reference value: 1.1–3.2 × 10^9^/L). Cerebrospinal fluid assessment showed an albuminocytologic dissociation with increased protein level (98 mg/dL, reference value: 8–43 mg/dL) and normal cell count (2 × 10^6^/L, reference value: 0–8 × 10^6^/L). Additional serological tests (i.e., ANA, anti-DNA, c-ANCA, p-ANCA, Campylobacter jejuni, Mycoplasma pneumoniae, *Salmonella enterica*, Cytomegalovirus, Epstein-Barr virus, herpes simplex virus 1 and 2, Varicella-Zoster virus, influenza virus A and B, human immunodeficiency virus) were negative. Normal serum vitamin B12 level and serum protein electrophoresis were found.

Four days after neurological symptoms and signs onset, motor nerve conduction studies showed delayed distal latencies and absent *F* waves in early course, supporting demyelinating pattern in accordance with GBS diagnostic criteria (Table 1). Sensory nerve conduction showed nerve action potentials; the recorded values were all in range, in line with the patient clinic.

**Table 1:** This table summarizes the main anamnestic and clinical patient information contained in all the studies published to date on GBS in COVID-19 patients

Authors/month-year	Method	Number of cases	Age (years)	Sex	GBS subtype	GBS-SARS-CoV-2 temporal relation
Zhao H. et al. [11], April 2020	Case report	1	61	Female	AIDP	Para-infectious
Sedaghat Z. et al. [12], April 2020	Case report	1	65	Male	AIDP	Post-infectious
Ottaviani D. et al. [10], April 2020	Case report	1	66	Female	Mixed AIDP and AMSAN	Post-infectious
Alberti P. et al. [13], April 2020	Case report	1	71	Male	AIDP	Post-infectious
Padroni M. et al. [14], April 2020	Case report	1	70	Female	AIDP	Post-infectious
Camdessanche J.P. et al. [15], April 2020	Case report	1	64	Male	AIDP	Post-infectious
Virani A. et al. [14], April 2020	Case report	1	54	Male	NR	Para-infectious
Coen M. et al. [16], April 2020	Case report	1	70	Male	AIDP	Post-infectious
El Otmani H. et al. [15], April 2020	Case report	1	70	Female	AIDP	Para-infectious
Toscano G. et al. [17], May 2020	Case series	5	NR	NR	AMSAN in 3 cases and AIDP in 2 cases	Post-infectious
Scheidl E. et al. [18], May 2020	Case report and literature review	1	54	Female	AIDP	Post-infectious
Riva N. et al. [19], May 2020	Case report	1	60	Male	AIDP	Post-infectious
Assini A. et al. [20], May 2020	Case series	2	55 and 60	Both male	AIDP and AMSAN	Both para-infectious
Bigaut K. et al. [21], May 2020	Case series	2	48 and 70	Male and female	Both AIDP	Both post-infectious
Romero-Sánchez C. M. et al. [22], June 2020	Case report	1	NR	NR	NR	NR
Chan J.H. et al. [23], June 2020	Case report	1	58	Male	AIDP	Para-infectious

*AIDP* acute inflammatory demyelinating polyradiculoneuropathy, *AMSAN* acute motor and sensory axonal neuropathy, *COVID-19* coronavirus disease 2019, *GBS* Guillain-Barré syndrome, *NR* not reported

A diagnosis of Guillain-Barré syndrome was then made. Intravenous immunoglobulin was administered at a dose of 0.4 g/kg for 5 days. Thrombocytopenia and lymphocytopenia progressively returned in the following days, with complete resolution of the admission radiological pulmonary findings. The improvement of the respiratory and laboratory clinic was followed by a progressive recovery of limb strength and a return to osteo-tendon normoreflexia. Thirty days after hospitalization, following the negative result of the oropharyngeal swab for SARS-CoV-2, the patient was discharged to continue the rehabilitation program at home.

## Discussion

We described a case of acute progressive symmetric ascending flaccid tetraparesis in a COVID-19 patient with diagnosis of AIDP subtype of GBS. The anamnestic, clinical, electrophysiological, and laboratory evidence leads to a likely causal association with SARS-CoV-2.

Starting from the first case of GBS SARS-CoV-2 infection-related described by Zhao H. et al. [11], a series of cases have been reported in the literature, supporting the post-infectious and para-infectious etiopathological correlation between SARS-CoV-2 and this acute polyradiculoneuropathy (Table 2). A systematic review of the literature on GBS and its correlation with SARS-CoV-2 infection was performed. Multiple searches were made on PubMed and Scopus by cross-referencing the following keywords: “Guillan-Barrè”, “Guillan-Barrè syndrome”, “COVID-19”, “SARS-CoV-2”, “para-infectious”, “post-infectious”, “molecular mimicry”, “neuropathy”, “flaccid”, “polyradiculoneuropathy”, “ACE-2”, “pathogenesis”. Other pertinent articles were retrieved through reference analysis. Inclusion criteria were the report of GBS clinical manifestation in COVID-19 patients. To date, June 6th, 2020, 16 papers have been published regarding the GBS-SARS-CoV-2 correlation. Adding up all the cases reported in the literature, 23 COVID-19 patients with GBS have been described, including our case report. There was no gender prevalence, and the average age of the patients was 61 years. A slight prevalence of AIDP over AMSAN subtype was observed. Post-infectious cases were predominant over para-infectious cases.

**Table 2:** Motor nerve conduction study of peripheral nerve of lower limbs. Distal latencies and absent of *F* waves support the diagnosis of demyelinating pattern (i.e., AIDP GBS subtype)

Peripheral nerve stimulated	Side	Stimulation point	Recorded point	Distal latency (ms)	Amplitude (mV)	Conduction velocity m/s	F latency (ms)
Tibial	Left	Popliteal fossa	Abductor hallucis brevis	14,56 (rv ≤ 7,2)	6,11 (rv ≥ 4)	42 (rv ≥ 40)	/
		Ankle	Abductor hallucis brevis	9,78 (rv ≤ 5,1)	7,29 (rv ≥ 4)		
	Right	Popliteal fossa	Abductor hallucis brevis	16,89 (rv ≤ 7,2)	5,97 (rv ≥ 4)	44 (rv ≥ 40)	/
		Ankle	Abductor hallucis brevis	9,23 (rv ≤ 5,1)	6,55 (rv ≥ 4)		
Common peroneal	Left	Below fibula	Extensor digitorum brevis	13,89 (rv ≤ 7,5)	3,45 (rv ≥ 2)	45 (rv ≥ 42)	/
		Ankle	Extensor digitorum brevis	9,55 (rv ≤ 5,5)	2,64 (rv ≥ 2)		
	Right	Below fibula	Extensor digitorum brevis	15,61 (rv ≤ 7,5)	2,01 (rv ≥ 2)	43 (rv ≥ 42)	/
		Ankle	Extensor digitorum brevis	7,78 (rv ≤ 5,5)	2,98 (rv ≥ 2)		

*rv* reference value

GBS is an acute flaccid paralytic disease that most commonly presents with progressive symmetric weakness and areflexia. GBS usually occurs following a respiratory or gastrointestinal infection, with a presentation latency varying between 3 days and 6 weeks [9]. The supposed pathophysiological mechanism is the “molecular mimicry”, an aberrant autoimmune response to a preceding infection which evokes a cross-reaction against the peripheral nerve antigens (e.g. production of anti-ganglioside antibodies in AMSAN GBS subtype preceded by Campylobacter jejuni infection) [9]. For this reason, GBS can be defined as para-infectious neurological disease [4]. SARS-CoV-2 nervous tissue damage can be both related to the direct neuroinvasive action (through direct binding with ACE-2 receptors) [24] and to an indirect injury of the immune system. In the latter case, the mechanism of immune-mediated damage can be due both to an overactivation of the immune system with hyperproduction of interleukin-6, and to the generation of an autoimmune reaction [18].

The infectious bacterial agents classically associated with GBS are Campylobacter jejuni, which is the most frequent, Mycoplasma pneumoniae, and *Haemophilus influenzae*; Cytomegalovirus, Epstein-Barr virus, and Influenza-A virus are the most involved viral pathogens [9]. Cases associated with hepatitis E virus infection and measle infection have also rarely been found [25, 26]. In 2015 and 2016, cases of GBS related to the Zika virus were also reported [10, 27]. In addition, recent evidence reported some cases of GBS in COVID-19 patients, to the point of speculating a possible association between the acute polyradiculopathy and SARS-CoV-2 infection [10–23, 28, 29].

Post-infectious refers to patients with GBS arisen once the SARS-CoV-2 infection has resolved, while para-infectious if GBS occurred during COVID-19 [14]. In our case, the respiratory clinic slightly preceded the appearance of neurological symptoms, while the pulmonary, laboratory and radiological positivity to SARS-CoV-2 accompanied the whole clinical course of GBS, to have an almost parallel resolution. These data are suggestive for a para-infectious mechanism.

As well described in the literature, GBS is an immune-mediated disorder due to a molecular mimicry mechanism [9]. However, a real verification of the production of specific antibodies against gangliosides present on the surface of the nerve myelin sheaths is still lacking [12]. For this reason, further studies are needed to better clarify the pathophysiological mechanism of GBS in patients with COVID-19.

The diagnosis of SARS-CoV-2-related GBS in our patient was supported by a series of laboratory findings associated with the incipient clinic and electrophysiological data. In particular, the serologies of the most common pathogens associated with GBS, such as Campylobacter jejuni, Mycoplasma pneumoniae, *Salmonella enterica,* Cytomegalovirus, Epstein-Barr virus, herpes simplex virus 1 and 2, Varicella-Zoster virus, influenza virus A and B, and human immunodeficiency virus, were negative. Antibody tests for GBS-associated autoimmune diseases (e.g., ANA, anti-DNA, c-ANCA, p-ANCA) were also negative. Furthermore, in support of GBS diagnosis, we observed a marked albuminocytologic dissociation. However, we cannot prove with certainty that a COVID-19 para-infectious AIDP subtype of GBS has occurred, as the sensitivity RT-PCR test of the oropharyngeal swab is suboptimal [14]. Furthermore, some rarer but endemic Northern Italy infectious agents that can be related to para-infectious GBS, such as West Nile virus and Toscana Virus, have not been tested [14]. Besides, adequate paraneoplastic screening was not performed, and antiganglioside antibodies were not studied. Despite this, based on the anamnestic, laboratory, neurophysiological, and clinical data collected, we can support the correlation between the onset of GBS and COVID-19, in line with emerged data of the literature review.

## Conclusion

While it is well known that SARS-CoV-2 correlates with respiratory and gastrointestinal manifestations, systemic and neurological involvement is still being studied. The case we described of GBS in a COVID-19 patient adds to those already reported in the literature, in support of SARS-CoV-2 triggering of GBS.

The aim of this work is to shed more light on the neurological manifestations of COVID-19, not as a corollary of classic respiratory and gastrointestinal symptoms, but as SARS-CoV-2-related standalone clinical entities. To date, it is essential for all specialists, clinicians, and surgeons, to direct attention towards the study of this virus, in order to clarify the spectrum of its neurological manifestations.
